# Structural phenotypes of knee osteoarthritis: potential clinical and research relevance

**DOI:** 10.1007/s00256-022-04191-6

**Published:** 2022-09-26

**Authors:** Frank W. Roemer, Mohamed Jarraya, Jamie E. Collins, C. Kent Kwoh, Daichi Hayashi, David J. Hunter, Ali Guermazi

**Affiliations:** 1 Quantitative Imaging Center, Department of Radiology, Boston University School of Medicine, 820 Harrison Avenue, FGH Building, 4th floor, Boston, MA 02118 USA; 2 Department of Radiology, Universitätsklinikum Erlangen and Friedrich-Alexander University Erlangen-Nürnberg (FAU), Maximiliansplatz 3, 91054 Erlangen, Germany; 3 Department of Radiology, Massachusetts General Hospital, Harvard University, 55 Fruit St, Boston, MA 02114 USA; 4 Orthopaedics and Arthritis Center of Outcomes Research, Brigham and Women’s Hospital, Harvard Medical, School, 75 Francis Street, BTM Suite 5016, Boston, MA 02115 USA; 5 University of Arizona Arthritis Center, The University of Arizona College of Medicine, 1501 N. Campbell Avenue, Suite, Tucson, AZ 8303 USA; 6 Department of Radiology, Stony Brook University Renaissance School of Medicine, State University of New York, 101 Nicolls Rd, HSc Level 4, Room 120, Stony Brook, NY 11794-8460 USA; 7 Department of Rheumatology, Royal North Shore Hospital and Institute of Bone and Joint Research, Kolling Institute, University of Sydney, Reserve Rd, St. Leonards, 2065 NSW Australia; 8 Department of Radiology, VA Boston Healthcare System, 1400 VFW Parkway, Suite 1B105, West Roxbury, MA 02132 USA

**Keywords:** Osteoarthritis, Phenotypes, Structure, MRI, Clinical trial

## Abstract

A joint contains many different tissues that can exhibit pathological changes, providing many potential targets for treatment. Researchers are increasingly suggesting that osteoarthritis (OA) comprises several phenotypes or subpopulations. Consequently, a treatment for OA that targets only one pathophysiologic abnormality is unlikely to be similarly efficacious in preventing or delaying the progression of all the different phenotypes of structural OA. Five structural phenotypes have been proposed, namely the inflammatory, meniscus-cartilage, subchondral bone, and atrophic and hypertrophic phenotypes. The inflammatory phenotype is characterized by marked synovitis and/or joint effusion, while the meniscus-cartilage phenotype exhibits severe meniscal and cartilage damage. Large bone marrow lesions characterize the subchondral bone phenotype. The hypertrophic and atrophic OA phenotype are defined based on the presence large osteophytes or absence of any osteophytes, respectively, in the presence of concomitant cartilage damage. Limitations of the concept of structural phenotyping are that they are not mutually exclusive and that more than one phenotype may be present. It must be acknowledged that a wide range of views exist on how best to operationalize the concept of structural OA phenotypes and that the concept of structural phenotypic characterization is still in its infancy. Structural phenotypic stratification, however, may result in more targeted trial populations with successful outcomes and practitioners need to be aware of the heterogeneity of the disease to personalize their treatment recommendations for an individual patient. Radiologists should be able to define a joint at risk for progression based on the predominant phenotype present at different disease stages.

## Introduction

The public health burden of osteoarthritis (OA) is substantial. It is the most common form of arthritis and is increasing in prevalence over time [1]. The disability and costs related to the disease are high [2–4]. Current treatments for the condition are limited to exercise, self-management programs, analgesics, and eventually total joint replacement [5]. Therefore, there is an urgent need to better understand the underlying etiology of this disease and to identify effective treatments and preventative strategies for the condition. Successfully alleviating pain and halting or minimizing the progression of joint damage in patients with knee OA has been a challenge for many years. This has been partially attributed to the heterogeneity of the disease, which poses challenges when developing a one-size-fits-all therapy for a heterogeneous and unselected patient population [6–8]. OA may occur due to a wide variety of factors (e.g., post-traumatic, genetic, metabolic, biomechanical), and multiple mechanisms can contribute to pain perception. Similarly, there is considerable variability in the trajectory of the disease prognosis, with some individuals experiencing progression, while others remain stable for many years [9–12]. In this context, it has been proposed that OA is a syndrome comprised of multiple distinct phenotypes rather than a single disease [13]. This narrative review will provide background on the concept of OA phenotypes and focus on the potential relevance of defining and understanding structural phenotypes based on imaging.

## The concept of OA phenotyping

Analogous to what has been described in the field of other heterogeneous conditions, an OA phenotype can be defined as a single or collection of disease characteristic(s) that describes differences between patients as they relate to distinct relevant outcomes (e.g., the severity of symptoms, prognosis, and response to treatment) [14]. Some reasons support the rationale for defining OA phenotypes. Firstly, from an epidemiologic perspective, it would be advantageous to clearly understand the entities that contribute to the development of different subtypes of OA [15]. Further, from a therapeutic perspective, identifying precise OA phenotypes would allow targeted treatments for specific subgroups and, ultimately, the identification of more efficacious treatments through successful clinical trials [6]. Previous studies have grouped knee OA patients into distinct phenotypes from different perspectives [16–20]. They have used different sets of characteristics to determine the phenotypes (e.g., imaging findings, biochemical profiles, clinical features) and used either outcome-based definitions (e.g., trajectories of clinical or structural progression) or definitions based on baseline characteristics with a subsequent association of the phenotypes with outcomes [21]. In addition, various distinct analytical methods have been used to identify phenotypes [7, 13, 15, 22]. At present, however, there is a lack of clarity over the phenotypes that may comprise the disease of OA [23]. A systematic review on OA phenotypes by Deveza and colleagues found substantial heterogeneity across the studies in the selection of participants and in the characteristics and methods used to investigate knee OA phenotypes [24]. Predominantly, cross-sectional evidence suggests that pain sensitization, psychological distress, radiographic severity, body mass index (BMI), muscle strength, inflammation, and comorbidities, particularly metabolic syndrome, play a part in distinguishing clinically distinct phenotypes. In addition, gender, obesity and other metabolic abnormalities, the pattern of cartilage damage, and inflammation may be implicated in delineating structural knee OA phenotypes. The authors suggest that patient and disease characteristics (possibly reflecting different disease stages) should be considered when phenotyping knee OA patients [24]. Another systematic review on OA phenotypes conducted by Dell’Isola et al. reviewed 24 studies that proposed clinical phenotypes of knee OA and concluded there is evidence suggesting the existence of six mechanistically distinct phenotypes [25]: (1) chronic pain; (2) inflammatory mechanisms; (3) metabolic mechanisms of bone and cartilage local to the joint; (4) metabolic syndrome; (5) mechanical overload; and (6) and minimal joint disease. Although their proposed phenotypes are similar to the OA characteristics identified by Deveza et al., the authors acknowledged that it is uncertain if the six proposed phenotypes are distinct entities or if there is an overlap between them [25].

Although seemingly plausible, a wide range of views exists on how best to operationalize the concept of OA phenotyping. A recent multi-author project led by van Spil aimed to provide consensus-based definitions and recommendations that together create a framework for conducting and reporting OA phenotype research [26]. Four Delphi rounds were performed to achieve sufficient agreement on definitions and statements. OA phenotypes were defined as subtypes of OA that share distinct underlying pathobiological and pain mechanisms and their structural and functional consequences. The authors concluded that OA phenotypes are subtypes of OA that share distinct underlying pathobiological and pain mechanisms and their structural and functional consequences [26]. It has to be noted that regarding structural phenotypes, no such consensus is available today. A detailed summary of this Delphi exercise is provided in Appendix 1.

## Structural OA phenotypes: what do we know?

Multiple joint tissues may exhibit pathological changes, resulting in many potential targets for treatment. Consequently, it is unlikely that a single treatment for OA will be similarly efficacious in preventing or delaying the progression of all types of structural OA. Several authors are suggesting that OA comprises multiple phenotypes or subpopulations, defined based on the pathophysiology and structural manifestations of the disease [24, 27, 28]. These phenotypes may be characterized by specific clinical features, laboratory parameters, biochemical markers, and/or imaging criteria [24]. Regarding structure, the field in particular has focused on an articular cartilage phenotype [29], and more recently, a bone-driven cartilage progression phenotype has been suggested [30]. However, inflammation is recognized as being a central part of the OA pathology [31]. While inflammation may not be the primary initiator of disease, it is also present in the early stages of OA [32, 33] and may at some point be the driver of disease progression [34]. As multiple tissues are affected, it seems unlikely that all OA patients would be effectively treated with the same interventions. It is plausible that the failure, in part, of numerous phase II/III OA clinical trials, such as iNOS [35], bisphosphonates [36], and calcitonin [37], the partial failure of strontium ranelate [38], and potentially also the missing translation of structural improvement to clinical benefit in the FORWARD trial [39] have been due to the failure to identify specific patient subpopulations with structural abnormalities that matched the pharmacodynamics of the drug under study [22].

### Applicability of structural phenotyping in a DMOAD context

Due to the limitations in clinical trial duration, disease-modifying osteoarthritis drug (DMOAD) efficacy may be most efficiently evaluated in patients at risk of rapid progression of OA, which could be termed a fast progressor phenotype. Researchers are working to understand which joints are at risk of such an accelerated disease evolution [40–42]. Riddle et al. evaluated a prediction tool for estimating the probability of incident rapidly progressing radiographic knee OA in the following 4–5 years [43]. Persons with contralateral knee OA, a baseline OA grade of KL1, higher body mass index, and higher baseline Western Ontario and McMaster Universities arthritis index total scores were more likely to develop K&L grade of 3 or 4 within 5 years. Authors termed this the “incident tibiofemoral osteoarthritis with rapid progression phenotype” [43]. In addition to such accelerated progression phenotypes, additional structural phenotypes of OA have been proposed: an inflammatory phenotype; a subchondral bone phenotype, characterized by marked subchondral bone changes; a meniscal or cartilage-meniscus phenotype, which results in altered biomechanics and subsequent cartilage loss; and hypertrophic and atrophic phenotypes, characterized by the presence of large osteophytes or absence of osteophyte formation [28, 44, 45]. Of these phenotypes, only the last two can be adequately characterized using radiography.

**Fig. 1:**
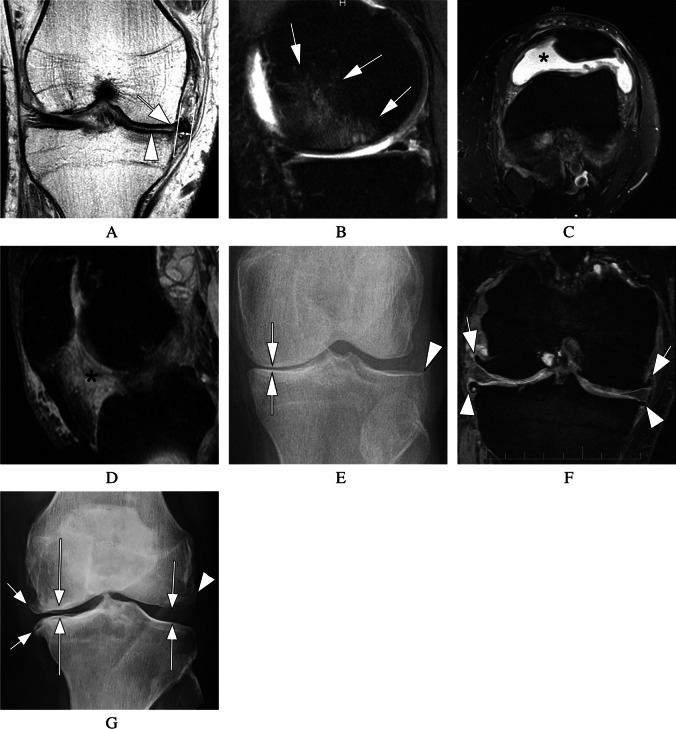
Phenotypic characterization. **A** The cartilage/meniscus phenotype is characterized by severe meniscal damage depicted in this example as partial meniscal maceration of the medial meniscal body (arrow) and is commonly associated with severe cartilage loss (arrows point to diffuse superficial cartilage damage of the medial tibia). There is severe meniscal extrusion (parallel lines and double-headed small arrow). In addition, there is diffuse superficial cartilage damage at the medial femur. Different definitions of a cartilage-meniscus phenotype have been proposed depending on the amount of cartilage damage and meniscal involvement (Roemer FW et al., Patterns of progression differ between Kellgren-Lawrence 2 and 3 knees fulfilling different definitions of a cartilage-meniscus phenotype in the Foundation for National Institutes of Health Osteoarthritis Biomarkers study (FNIH). Osteoarthritis and Cartilage Open 2022;4(3): 100284.). Depending on the definition, this knee may also fulfill the imaging requirements of an atrophic phenotype, characterized by advanced cartilage loss and little or no marginal osteophytes. **B** Bone phenotype. A large bone marrow lesion (BML) is present in the medial central subregion of the medial femur (grade 3, arrows). The size of the BML defines this knee as a bone phenotype. **C** The inflammatory phenotype is characterized by severe joint effusion-synovitis (asterisk). **D** So-called Hoffa-synovitis, a non-specific surrogate of whole knee synovitis, is another manifestation of inflammation and is considered for the classification of an inflammatory structural phenotype. **E** The atrophic phenotype is characterized by severe cartilage loss without relevant osteophyte formation. It can be diagnosed by radiography. Anterior–posterior radiograph shows severe medial joint space narrowing (arrows), defining this knee as Kellgren-Lawrence grade 3. There is, however, only a tiny, equivocal osteophyte at the lateral tibia (arrow). The discrepancy between joint space narrowing (a surrogate for cartilage and meniscal damage, and meniscal extrusion) defines this knee as having an atrophic phenotype. **F** The hypertrophic phenotype is characterized by large osteophytes with only minor cartilage loss and can be defined either radiographically based on joint space narrowing or by MRI based on cartilage integrity or damage. Coronal dual echo at steady state (DESS) reformatted image shows large osteophytes at the medial and lateral femoral joint margin (arrows) and moderate-sized osteophytes at the medial and lateral tibia (arrowheads). **G** Example of the hypertrophic phenotype as visualized by X-ray. Large marginal osteophytes are seen at the medial compartment (short arrows) but also laterally (arrowhead). The medial and lateral joint space is largely preserved (long arrows)

Acknowledging that these subtypes may not necessarily fulfill the overarching suggested definition of OA phenotypes, including clinical parameters [26], structural stratification may be relevant, particularly in the context of clinical trials. The recently introduced Rapid OsteoArthritis MRI Eligibility Score (ROAMES) system allows structural classification based on abbreviated MRI assessment and, thus, may potentially be applicable in screening efforts for inclusion in DMOAD trials [46]. In a recent report based on the Foundation for National Institutes of Health (FNIH) Osteoarthritis Biomarkers Consortium cohort, we found that the bone phenotype was associated with increased odds of progression, while the inflammatory and cartilage-meniscus phenotypes were not [47]. However, a drawback of that analysis was the low prevalence of the phenotype yield using the primary definitions according to ROAMES. This was the case, particularly for the inflammatory and cartilage/meniscus phenotypes (prevalence < 5% for both phenotypes) [46]. Illustrative examples of the different MRI-defined structural phenotypes are shown in Fig. 1. These phenotypes exhibit distinct structural characteristics, such as the absence or presence of osteophytes in atrophic or hypertrophic phenotypes, or structural characteristics predisposing a joint to faster disease progression. This concept has clear limitations because structural phenotypes overlap, and more than one could be present in an individual. OA is a heterogeneous disease with different pathways involving multiple tissues that exhibit structural damage. For this reason, characterizing a predominant structural phenotype has been suggested [48]. Definition of these phenotypes will undoubtedly need to be refined further in the future, and novel analytical approaches could help in doing so [21, 24, 49].

### Inflammatory phenotype

From an MRI perspective, the inflammatory phenotype is characterized by marked synovitis and/or joint effusion on MRI [46]. Synovitis in OA is thought to be a secondary phenomenon related to cartilage deterioration, and synovitis also seems to play a role in the progression of cartilage loss in knee OA [50, 51]. Using arthroscopy as a reference standard to evaluate synovial abnormalities, Ayral et al. [52] described that 29% of knees with OA had a reactive aspect (a proliferation of opaque villi), and 21% had an inflammatory aspect (hypervascularization of the synovial membrane and/or proliferation of hypertrophic and hyperemic villi). Of note, an association with progressive cartilage damage at the 1-year follow-up visit was found only in the group with inflammatory synovitis [52]. A recent meta-analysis showed that static and dynamic contrast-enhanced (CE)-MRI evaluation of knee synovitis were positively correlated with macroscopic and microscopic markers of synovial membrane inflammation. Among the features of synovial tissue inflammation, CE-MRI scores correlated best with the inflammatory infiltrates of synovial tissue [53]. Others have suggested that dynamic CE MRI parameters of synovial enhancement may be more sensitive to the early response to treatment and more strongly associated with changes in pain than only synovial volume and may be better outcomes for assessment of structural effects of treatment in OA [54]. In addition, dynamic CE MRI parameters have shown good reproducibility [55].

### Subchondral bone phenotype

The bone phenotype of knee OA is characterized by large bone marrow lesions (BMLs) in one or several compartments. BMLs are defined on fluid-sensitive fat-suppressed MRI sequences as non-cystic subchondral areas of ill-defined hyperintensity commonly seen together with cartilage damage in the same area [56, 57]. BMLs are important predictors of subsequent structural progression and symptom fluctuation in knee OA and have thus become a treatment target for novel therapeutic approaches [58–60]. Due to the fluctuating nature of BMLs and the possibility of regression, potential treatment effects have been shown for time intervals as short as 6–12 weeks [61, 62].

### Meniscus-cartilage phenotype

A meniscus-cartilage phenotype exhibits extensive meniscal damage and/or meniscal extrusion and wide-spread cartilage loss on MRI. Load distribution and shock absorption by the meniscus are crucial in protecting the tibiofemoral compartments. Meniscal morphology is rarely normal in knee compartments affected by OA; instead, the meniscus is often torn, shows substance defects, or is even destroyed [63]. Although extensive radiological literature on the different types of meniscal pathology is available, little emphasis has been placed on the relevance of these differences to incident OA or progression of the disease [64]. MRI-determined meniscal pathology, including meniscal extrusion, predicts cartilage loss in the tibiofemoral compartments [65–67]. The cartilage-meniscus phenotype is of particular relevance given the target tissue of most DMOAD approaches is cartilage—either by anti-catabolic or anabolic pathways. In a recent post hoc analysis based on the FNIH dataset, we showed that phenotypic stratification of the cartilage-meniscus phenotype into different subtypes is feasible and may help define various trial cohorts at screening. Increased odds for progression were seen for KL2 knees and all definitions, while a seemingly protective effect was seen for KL3 knees. The latter fact was explained by the fact that KL3 knees stratified by the suggested definitions have comparably mild cartilage damage at screening. In the same study, it was shown that one-third of knees with KL2 did not have any medial cartilage damage, which is an important finding and needs to be considered when selecting patients for inclusion into clinical trials based on only X-ray assessment [68]. While short-term worsening of cartilage damage has been described for time intervals as short as 6 months [68], treatment effects assessed by quantitative approaches are usually only observed after 12–24 months in a clinical trial setting [39]. However, clear recommendations regarding trial duration are not available as this depends on multiple factors, including eligibility criteria and the primary outcome of interest [69].

### Hypertrophic and atrophic phenotypes

A hypertrophic or atrophic OA phenotype is characterized by the presence or absence of osteophytes and respectively their size. A cross-sectional analysis of the population-based Framingham cohort focused on different phenotypes of knee OA on MRI and demonstrated that severe cartilage damage in the knee is commonly associated with large osteophytes [44]. However, osteophyte formation may follow cartilage loss, in which case OA might manifest as an atrophic OA phenotype characterized by an absence of osteophytes or only tiny osteophytes but presence of marked cartilage loss. Using a stringent MRI-based definition of atrophic knee OA, such a phenotype has a very low prevalence in the general population [44]. Currently, no definition of atrophic OA based on radiography is available with this entity commonly being identified by definite joint space narrowing without any osteophytes or by a marked discordance between joint space narrowing and the size of associated osteophyte formation. A study based on the Multicenter Osteoarthritis Study (MOST) cohort showed, surprisingly, that the progression of joint space narrowing and cartilage loss was less commonly associated with the atrophic phenotype of knee OA (based on radiographic and MRI definitions) compared to non-atrophic knee OA [45].

### Additional aspects

In addition to these five distinct structural phenotypes, features visible on MRI, such as meniscal extrusion and prevalent meniscal structural damage, BMLs, and prevalent cartilage lesions in knees at baseline—or combinations thereof—could be used to identify a subpopulation at high risk of progressive cartilage loss within a short time interval. Although measurable quantitative cartilage loss over 6 months is thought to be rare, data from the Joints on Glucosamine Study suggest that cartilage loss, as well as the development or progression of BMLs and meniscal extrusion, does occur within this time frame [70], suggesting that some of these MRI-detected structural changes could be used as outcomes in a well-chosen study population. In addition, in the MOST cohort, the presence of a high BMI, meniscal damage, synovitis or effusion, or any severe MRI-detectable lesions at baseline were strongly associated with an increased risk of rapid cartilage loss over a 30-month period [41], which suggests that assessment of such features could enable the selection of a trial population at high risk of disease progression. Although it is not known whether knees at increased risk of disease progression would be more likely to benefit from a pharmacological intervention than others, including such patients in clinical trials, could increase the efficiency of the trials by decreasing their overall duration, providing that the intervention was sufficiently potent. Thus, these MRI-based risk factors could be used to select individuals for inclusion in trials of preventive or therapeutic interventions for OA, depending on the trial length.

Recently, it has been suggested that those patients most likely to benefit from structure modification are those that on one-hand exhibit specific imaging characteristics as described and are also at increased genetic risk for OA incidence or progression. In a recent pilot study, Lo et al. sought to assess the potential of studying the offspring of people with and without knee osteoarthritis in order to understand the risk factors and heritability for knee osteoarthritis. The authors found that radiographic tibiofemoral osteoarthritis and meniscal abnormalities were more common among offspring with parental osteoarthritis status than those without. By selecting patients with bilateral medial radiographic tibiofemoral knee osteoarthritis, likely a heritable component of the disease, increased risk for OA progression may be likely. The data of this study supported this hypothesis as 8 out of 9 offspring of probands with OA were reported to have bilateral knee osteoarthritis [71].

## Limitations

**Fig. 2:**
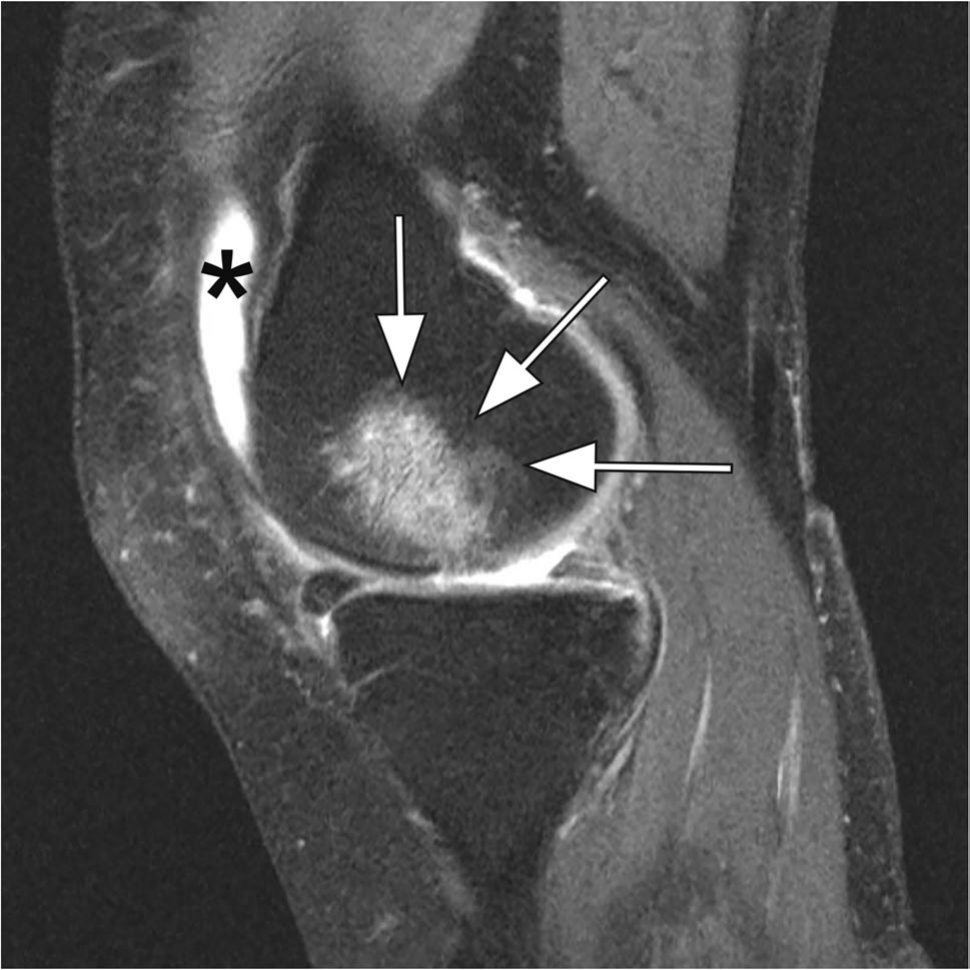
Structural phenotypes may overlap and one joint may exhibit more than one phenotype. Sagittal intermediate-weighted fat suppressed image shows a large bone marrow lesion at the central subregion of the medial femur (arrows). In addition, there is a moderate-to-large joint effusion-synovitis (asterisk). This knee fulfills the definition for two phenotypes, i.e., the subchondral bone and the inflammatory phenotype

We acknowledge that the MRI-based attempts at structural phenotyping have limitations because structural phenotypes are likely overlapping and rarely mutually exclusive, with more than one phenotype may be present in an individual depending on the stage of the disease as exemplified in Fig. 2. Further, these phenotypes are based on a priori hypotheses; whether other relevant structural phenotypes may exist has not been agnostically evaluated. We also acknowledge that structural features are only one aspect that drives disease progression and may define an OA phenotype. In the mentioned systematic review on OA phenotypes, Deveza and colleagues found significant heterogeneity across studies in the selection of participants and characteristics and methods used to investigate knee OA phenotypes [24]. Pain sensitization, psychological distress, radiographic severity, BMI, muscle strength, systemic inflammation, and comorbidities also play a part in distinguishing clinically distinct phenotypes as opposed to structural phenotypes. In addition, sex, obesity, other metabolic abnormalities, genetic factors, and the pattern of cartilage damage and inflammation may be implicated in delineating structural knee OA phenotypes. A lack of studies investigating structural phenotypes and disease progression has been clearly acknowledged [24].

## The road ahead

In summary, structural OA phenotyping is still in its infancy. Several a priori definitions for different OA phenotypes have been suggested, but these need further exploration and validation. Structural phenotyping should be part of eligibility screening in DMOAD trials, and there needs to be a realization that radiography alone cannot characterize a joint based on the predominant joint tissue involved in the disease process. Hopefully, the suggested structural phenotypic stratification may result in more targeted trial populations and will eventually decrease the numbers of participants included in DMOAD trials. Individuals should be included based on the potential specific mode of action of a given pharmacological compound, with the ultimate result of a successful trial that demonstrates the efficacy of the compound under study. Given a specific phenotype exists and has been included to a clinical trial, the outcome measure of a specific therapy needs to be tailored. A clinical trial testing, e.g., pharmacologic compound that is targeting the subchondral bone, should include subchondral bone measures as an outcome. A trial that is investigating the efficacy of an anti-inflammatory compound needs to include structural measures of inflammation in order to be able to quantify changes from baseline to later follow-up time points, i.e., be able to measure treatment effects. Practitioners need to be aware of the heterogeneity of the disease to personalize their treatment recommendations for individual patients, and radiologists should be able to define a joint at risk for progression based on the predominant structural phenotype present at different disease stages.

## Data Availability

Not applicable.

## Appendix 1. Final consensus statements on OA phenotypes according to van Spil et al.^1^

1. OA phenotypes are subtypes of OA that share distinct underlying pathobiological and pain mechanisms and their structural and functional consequences.
2. OA phenotypes can become apparent in differences in risk factors, prognostic factors, nature and extent of symptoms and signs, disease trajectory, and/or responsiveness to particular treatments or treatment in general.
3. An OA phenotype classification system is likely to consist of input variables that together reflect (the likelihood of) the presence of one or more pathobiological and pain mechanisms.
4. Classification systems are likely to use one or more measures from either one or more domains (e.g., imaging markers, biochemical markers, and pain) to identify a clinically relevant OA phenotype or phenotypes.
5. The potentially identified phenotype(s) should differ from others in terms of clinically relevant disease-driving factors and/or outcomes.
6. Research efforts may initially lead to multiple proposed phenotype classification systems. Eventually, these should be aligned and come together in one.
7. 7a Differences in the disease stage may cause different results from OA phenotyping studies between study populations. It is likely that the nature and course of disease stages may differ between patients and phenotypes.
  7b Disease stage(s) of the study population should always be reported. Reasons to take or not take disease stage into account in the analyses (e.g., to adjust for confounding or look for interaction) should be weighted for every study.
8. 8a Some components of pathobiological and pain mechanisms in OA may be similar between different joints such as knee and hip (e.g., synovitis, central pain perception), while others may differ (e.g., menisci, femoral head shape). The decision to extrapolate findings from one joint to another, or not, should be justified.
  8b Phenotype classification systems can be designed for individual joints or systemically (e.g., for multiple joints in one patient), depending on the pathobiological and pain mechanism that is under study and the goal of the study.
9. Data-driven approaches for constructing phenotype classification systems are generally preferable over expert opinion-based approaches, as long as they are performed using high-quality data and appropriate statistics, are reproducible, and have clinical validity, relevance, and applicability as judged by experts in the field.
